# Invasive Fish and Sea Urchins Drive the Status of Canopy Forming Macroalgae in the Eastern Mediterranean

**DOI:** 10.3390/biology12060763

**Published:** 2023-05-24

**Authors:** Athanasios Nikolaou, Konstantinos Tsirintanis, Gil Rilov, Stelios Katsanevakis

**Affiliations:** 1 Department of Marine Sciences, University of the Aegean, 81100 Mytilene, Greece; 2 National Institute of Oceanography, Israel Oceanographic and Limnological Research (IOLR), Haifa 31080, Israel; 3 The Leon H. Charney School of Marine Sciences, Marine Biology Department, University of Haifa, Mt. Carmel, Haifa 31905, Israel

**Keywords:** canopy-forming macroalgae, marine forests, *Cystoseira sensu lato*, herbivory, tropicalization, invasive species

## Abstract

**Simple Summary:**

The Mediterranean marine forests of canopy brown algae are important habitats that have been in decline in recent decades. This study examines the distribution and status of macroalgal communities in relation to the populations of the main herbivores (fish and sea urchins) in the eastern Mediterranean. In the warmer South Aegean and Levantine Sea, invasive herbivorous fish seem to drive canopy algae towards shallower waters, while native sea urchins have become rare, indicating population collapses. In the North Aegean, macroalgal forests were observed in intermediate depths, and native sea urchins still thrive and appear to exert grazing pressure on macroalgal forests in shallower waters. Our results provide useful information for policymakers and help guide future research and conservation efforts.

**Abstract:**

Canopy-forming macroalgae, such as *Cystoseira sensu lato*, increase the three-dimensional complexity and spatial heterogeneity of rocky reefs, enhancing biodiversity and productivity in coastal areas. Extensive loss of canopy algae has been recorded in recent decades throughout the Mediterranean Sea due to various anthropogenic pressures. In this study, we assessed the biomass of fish assemblages, sea urchin density, and the vertical distribution of macroalgal communities in the Aegean and Levantine Seas. The herbivore fish biomass was significantly higher in the South Aegean and Levantine compared to the North Aegean. Very low sea urchin densities suggest local collapses in the South Aegean and the Levantine. In most sites in the South Aegean and the Levantine, the ecological status of macroalgal communities was low or very low at depths deeper than 2 m, with limited or no canopy algae. In many sites, canopy algae were restricted to a very narrow, shallow zone, where grazing pressure may be limited due to harsh hydrodynamic conditions. Using Generalized Linear Mixed Models, we demonstrated that the presence of canopy algae is negatively correlated with the biomass of the invasive *Siganus* spp. and sea urchins. The loss of *Cystoseira s.l.* forests is alarming, and urgent conservation actions are needed.

## 1. Introduction

Macroalgal forests are important and prominent habitats of the Mediterranean Sea’s rocky reef ecosystems. Such forests are mostly dominated by bushy brown algae and can cover a substantial portion of the rocky intertidal and subtidal zones in many regions of the Mediterranean basin [1,2,3]. These mostly perennial macroalgae serve as ecosystem engineers, creating structurally complex communities that provide shelter and food, making them permanent homes and nursery habitats for numerous organisms. This enhances the biodiversity and productivity in coastal areas [4,5,6]. Macroalgal forests are fundamental to coastal ecosystem functioning, contributing to primary productivity, and supporting various provisioning, regulating (a potential for Blue Carbon) and cultural ecosystem services including food provision, ocean nourishment, climate regulation, life cycle maintenance, recreation and tourism [1,7,8,9] retaining high natural capital [10].

In Mediterranean rocky reefs, the most prominent canopy-forming perennial macroalgae are Fucales species of the genus *Cystoseira sensu lato*, which includes the genera *Cystoseira*, *Gongolaria* and *Ericaria* [11], or of the genus *Sargassum*. *Cystoseira s.l.* is an important indicator of ecological status in the context of the European Water Framework Directive (WFD 2000/60/EC) [12]. Moreover, *Cystoseira s.l*. is listed as strictly protected in Annex I of the Bern Convention (Council of Europe, 1979), and some species are listed in Annex II of the Barcelona Convention. Photophilic communities with canopy-forming algae in Mediterranean infralittoral and upper circalittoral rocky reefs, dominated by *Cystoseira s.l*., have been assessed as an endangered habitat [13].

Alarming documentation of declining *Cystoseira s.l.* forests has been reported in the Mediterranean Sea over the past few decades [14,15,16,17,18,19,20]. These declines result from both direct anthropogenic pressures, such as water quality decrease and habitat destruction [21,22,23,24,25], and indirect anthropogenic pressures, such as climate change through global warming and ocean acidification [26,27] and overgrazing by herbivores (mainly in the southeast basin due to biological invasions of rabbitfish or trophic cascades caused by overfishing that lead to an increase in sea urchin populations) [28,29,30,31]. It is essential to prioritize effective management actions to halt the loss of Mediterranean marine forests and conserve these keystone rocky reef habitats. Recently, canopy restoration methods have been developed and proposed as a management tool for the conservation of *Cystoseira s.l.* forests in the Mediterranean Sea [32,33,34,35,36,37].

Herbivory is a crucial factor that affects the structure of marine benthic primary producers [38]. In the Mediterranean, the sea urchins *Paracentrotus lividus* and *Arbacia lixula* have been identified as the primary invertebrate grazers that regulate macroalgal vegetation [39,40,41,42,43,44,45]. The indigenous herbivorous fish *Sarpa salpa* can also significantly deplete canopy-forming fucoid macroalgae [46,47], potentially affecting restoration efforts [48]. Furthermore, omnivorous fish such as *Diplodus vulgaris* may also act as grazers when attempting to feed on the resident macrofauna of macroalgal communities [49]. In the southeastern Mediterranean basin, the tropical invasive rabbitfishes *Siganus luridus* and *S. rivulatus* have become a significant part of the fish community [19,30,50,51], contributing to the formation of a novel eastern Mediterranean marine food web [52]. Their grazing impact on macroalgal communities has been detrimental, leading to the formation and maintenance of extensive barrens or depauperate turf-dominated communities [30,31,53,54]. At the easternmost edge of the Mediterranean, populations of the sea urchin *P. lividus* are collapsing due to elevated water temperatures caused by climate change [31,55]. Acting in synergy with ocean warming, the invasive *Siganus* are introducing further adverse effects on *P. lividus* [31] and *S. salpa* [56] through competition for shared resources. Due to their negative effects on Mediterranean ecosystems, both *Siganus* species have been assessed as among the worst invasive species to have been introduced to the Mediterranean basin [57,58]. Canopy-forming species distribution is also determined by abiotic parameters including the level of wave exposure [46,59,60], temperature [60,61], salinity [60] and depth. Depth, in particular, is strongly correlated with solar radiation, which affects the lower distribution limit of the forests [60,62]. Light penetration across the water column increases towards the southeastern Mediterranean [63]. Nevertheless, most observations of deep macroalgal forests, reaching depths of up to 40–50 m, have been reported primarily in the western Mediterranean [64,65,66]. Occasional records of Fucales reaching similar depths have been reported from the eastern Mediterranean [67]. Nonetheless, our knowledge of the distribution of deep macroalgal forests, particularly in the eastern Mediterranean, remains largely unknown.

In this study, we examined the ecological status of macroalgal communities and sea urchin density across various depth zones in the Levantine Sea (Cyprus and Israel) and the Aegean Sea (Lesvos, Skyros and Crete Islands). We also investigated the differences in fish assemblage biomass between sites as a potential corollary. All selected sites were dominated by rocky reefs and had at least some coverage of *Cystoseira s.l*. The sites were chosen along a gradient of climate change and biological invasions, ranging from the highly impacted Levantine Sea to the less impacted northern Aegean. We hypothesized that in the northern Aegean sites, which are cooler and less invaded, alien dominance will be lower and the status of the native grazers and algal forests will be better.

## 2. Materials and Methods

### 2.1. Study Area

The study area included the Aegean Sea (Greece) and the Levantine Sea (Cyprus and Israel) in the eastern Mediterranean Sea. The Mediterranean Sea’s biotic and abiotic compartments are geographically heterogeneous. It is characterized as a semi-enclosed concentration basin with high evaporation outfluxes, which dominate over freshwater inflows from land and precipitation. Atlantic surface waters enter the basin through the Gibraltar Strait and travel towards the eastern Mediterranean, where they become warmer and more saline due to intense evaporation. These waters reach the eastern Mediterranean where they sink and form the dense Levantine intermediate water, which travels back to the western Mediterranean to exit the basin through the Strait of Gibraltar [68]. The western and northern parts of the Mediterranean are characterized by colder waters with lower salinities and higher productivity. In contrast, the eastern Mediterranean is characterized by warmer, more saline, and more oligotrophic waters [69,70,71]. The tidal range in the Mediterranean Sea typically does not exceed one meter [72], which restricts intertidal habitats to relatively narrow zones. During winter, waves propagate towards the east and southeast throughout the basin, while in the summer, waves mainly propagate southwards from the Aegean and Levantine Seas [73].

**Figure 1:**
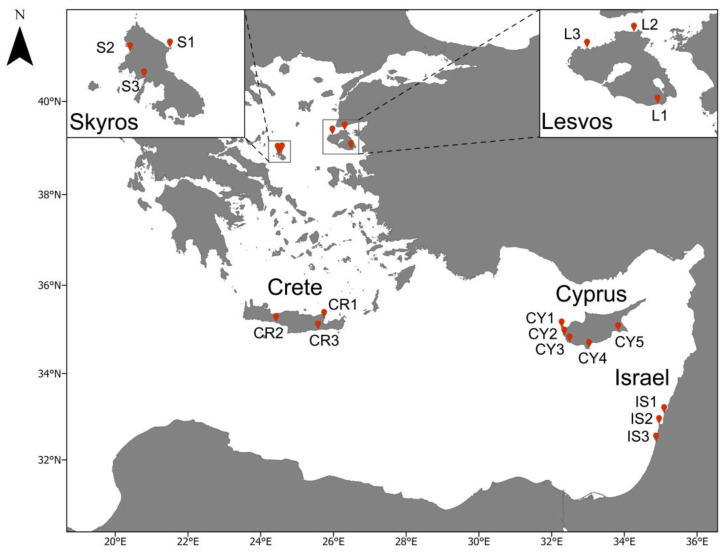
Sampling sites in the Aegean and the Levantine Sea, in the eastern Mediterranean. The sampling sites are labeled based on the respective sampling island.

In total, 17 sites were sampled across four different islands (Lesvos, Skyros, Crete, Cyprus) and the Israel coastline (Figure 1). Sampling sites were distributed across a temperature gradient (Table 1). Sampling at Crete and Skyros took place from late July to early August 2020, the Cyprus sites were sampled in early October 2020, and the Lesvos sites were sampled in November 2021 (except for L1, which was sampled in July 2020). The Israeli coast sites were sampled in spring 2021 or 2022. The sampling design in Israel differs from the other study sites as investigations were conducted by independent research teams, which consolidated their data post hoc.

### 2.2. Ecological Status of Macroalgal Communities

At each sampling site, the ecological status of the macroalgal communities was evaluated using the Ecosystem-Based Quality Index (reef-EBQI), developed by Thibaut et al. [75] that utilizes a scoring system to assess the rocky reef environmental status based on various ecological indicators, including the established macroalgal communities. The reef-EBQI framework was applied in five different depth zones (8 or 6, 5, 2, 1, 0–0.5 m) in most sampling sites. At depths of 8 (or 6 m if there were no reefs at 8 m), 5, 2 and 1 m, the macroalgal community status was assessed using photo-quadrats (50 × 50 cm) placed every 5 m along a 50 m transect line (sampling size: *n* = 10). A smaller photo-quadrat (25 × 25 cm) was used every 5 m at the depth zone of 0–0.5 m to enable the diver to take photo-samples under the effect of waves; by forming four osculated photo-samples of the smaller quadrat, the sampling surface remained the same as in deeper sampling zones. Surveys in Israel were conducted at depths of 2 and 7 or 8 m using a total of 15 quadrats (50 × 50 cm) per depth along a 30 m transect.

Each photo sample was categorized based on Thibaut et al.’s [75] scoring system (Table 2). The median of all status scores at each depth zone was used to assess the status score for that specific depth zone. The status score was determined by the dominant stratum present and its coverage. Five ordinal categories (from 0 to 4) were defined (Table 2).

### 2.3. Sea Urchins Densities Estimations

Sea urchin density (individuals m^−2^) was estimated at each studied depth zone by counting the sea urchins in ten 1 × 1 m quadrat frames along a 50 m transect line. The recorded species were the native sea urchins *P. lividus*, *A. lixula*, *Sphaerechinus granularis* and *Centrostephanus longispinus*, and the alien *Diadema setosum*. Sea urchins with test diameter < 3 cm were not considered in this study because they are challenging to detect and thus could result in high bias in density estimations [75]. In Israel, sea urchins were surveyed at 2 and 7 or 8 m depth, counting individuals at 50 × 50 cm quadrats (*n* = 15).

### 2.4. Fish Biomass Estimations

To estimate the fish biomass at each sampling site, a diver swam along a transect line in the 5 m depth zone, covering six replicated strips of 25 × 5 m at a constant speed. During the process, the diver recorded the encountered fish species and estimated the total length of each individual [76]. To mitigate any potential observer bias in length estimations, the observer underwent specialized training with depictions of fish silhouettes or objects of different sizes [77]. Fish biomass (g wet mass m^−2^) was estimated using the length–weight relationship from the available literature [78,79]. The fish were categorized into four trophic groups: planktivores, piscivores, invertivores and herbivores, based on Thibaut et al. [75]. The variability in fish biomass composition among sites was assessed using non-metric multidimensional scaling based on Bray–Curtis dissimilarity. Additionally, a cluster analysis was conducted to group the sites based on the Bray–Curtis dissimilarity. Non-metric multidimensional scaling and cluster analysis was performed using PRIMER-E v6 [80]. In Israel, fish were recorded in two 30 × 2 m transects: one at 2 m and the other at a 7 or 8 m depth.

### 2.5. Modelling Canopy Algae Presence

To investigate how herbivorous fish, sea urchins, and depth may partly explain the variability in the presence of perennial macroalgae, generalized linear mixed models (GLMMs) were constructed using the “lme4” R package [81]. The models used a binomial distribution and a logit link function [82]. The dependent variable (*Canopy*) was the presence of perennial macroalgae with more than 5% coverage (scores ≥ 3), which was recorded as a binary variable (*Canopy* = 1 if present, *Canopy* = 0 if absent). The independent variables included the biomass of alien (*AlienH*) and native (*NativeH*) fish herbivores, sea urchin density (*Su*) and depth (*Depth*). The site was considered a random variable, as samples within sites were dependent. The full model was:

$$\begin{array}{ll} Prob\left(Canopy_{i}=1\right)= & logit^{-1}(\beta_{0}+\beta_{1}AlienH_{i}+\beta_{2}NativeH_{i}+\beta_{3}Depth_{i}+\beta_{4}Su_{i}+a_{j}^{site})\\ & a_{j}^{site}\sim N\left(0,\;\sigma^{2}\right)\;for\;j=1,\ldots ,17 \end{array}$$

The probability of canopy presence for observation *i* depends on both fixed and random effects, which are assumed to be normally distributed with zero mean and a variance of *σ^2^*. We constructed sixteen candidate models using all possible combinations of independent variables (Supplementary Table S1). The most parsimonious model was selected based on the Akaike information criterion corrected for small samples (AICc). We estimated the variance inflation factor (VIF), using the “car” R package [83], to check for multicollinearity. No variable used had a VIF score greater than two, indicating low collinearity [84]. Model validation was conducted using the “Diagnostics for HierArchical Regression Models” (DHARMa) workflow, which involved inspecting scaled residuals simulated from fitted models [85]. To investigate the variation explained by individual independent variables, we calculated both the marginal R^2^ (variance explained by the fixed effects) and the conditional R^2^ (variance explained by both fixed and random effects) using the “partR2” package [86]. The 95% confidence intervals were obtained using parametric bootstrapping (1000 iterations). The packages “ggplot2” [87], “sjPlot” [88] and “egg” [89] were used for the visualization of the results. Analyses were carried out in R studio [90]. All maps were generated using ArcGIS Pro 2.6.0 software (https://www.arcgis.com accessed on 17 May 2023).

## 3. Results

### 3.1. Macroalgal Communities’ Ecological Status

**Figure 2:**
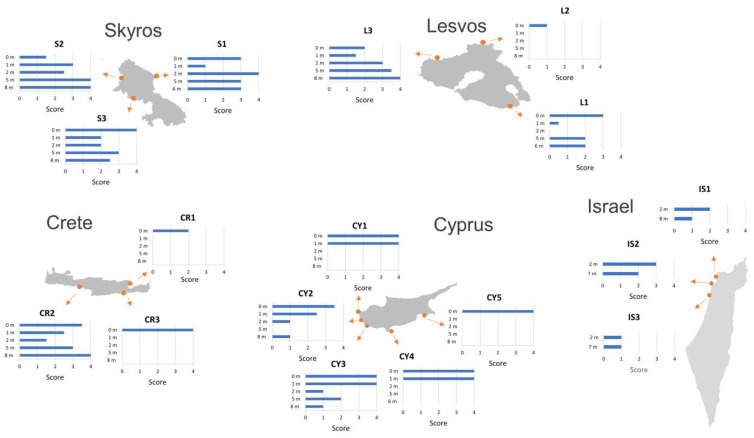
Ecological status scores of macroalgal communities based on Thibaut et al. [75] (0: very low, 1: low, 2: moderate, 3: good, 4: very good).

The reef-EBQI status scores of the macroalgal community revealed differing perennial vegetation bathymetric trends between the North Aegean sampling sites and the sampling sites of the South Aegean and Levantine Seas (Figure 2 and Supplementary Figure S1). In the North Aegean, most sampling sites (except L2) had good or very good status scores in at least one depth zone, indicating the presence of *Cystoseira s.l.* forests. In the southern sites, in Crete and Cyprus, high-status scores indicating high cover of *Cystoseira s.l.* were primarily observed in the shallower zones of 0–0.5 m and 1 m depth. In Israel, the status scores revealed an impoverished macroalgal community dominated by shrubby and turf algae, characterized by absent or sparse perennial fucoid vegetation, except for the shallow water in IS2 (the Shikmona coast in Haifa). The limited number of depth zones investigated in the Israeli sites did not allow for clear elaboration of the vertical distribution of macroalgae.

### 3.2. Sea Urchin Density

**Figure 3:**
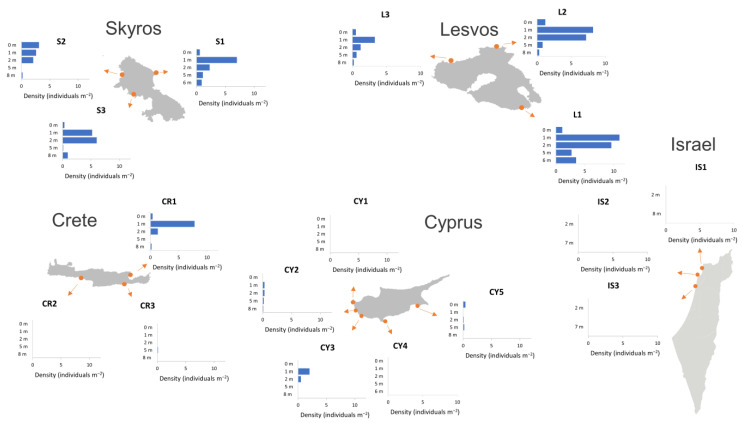
Sea urchin density (individuals m^−2^) at each study site for the different depth zones.

**Figure 4:**
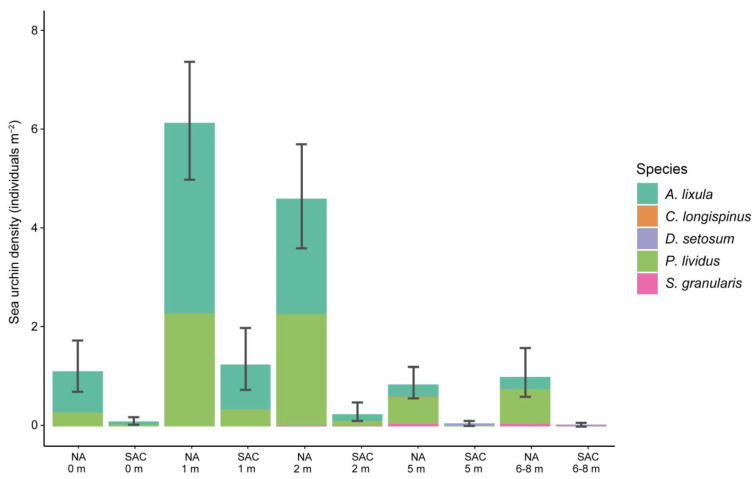
Mean sea urchin densities and 95% confidence intervals across the North Aegean sites (NA) and South AegeanCyprus sites (SAC) for different depth zones (bootstrapped results). The different colors in stacked bars represent the different sea urchin species recorded. The sea urchin density in Israel was zero; these records are not included in the mean values of the southern sites depicted here.

The sea urchin density estimations revealed the collapse of sea urchin populations in most of the southern sites of Crete, Cyprus and Israel (Figure 3). Sea urchins were absent or exhibited very low densities in all but one southern sampling site (CR1 in Crete) (Figure 3). In contrast, sea urchin populations in the North Aegean were abundant, with the highest densities recorded in the 1 and 2 m depth zones. In the North Aegean, the mean sea urchin density values and their 95% confidence intervals across the sites were 1.12 [0.70, 1.73], 6.13 [4.98, 7.37], 4.60 [3.60, 5.7], 0.85 [0.57, 1.20] and 1.00 [0.60, 1.58] individuals per m^2^ for the 0, 1, 2, 5 and 8 m depth zones, respectively (Figure 4). In most islands and depth zones, *A. lixula* and *P. lividus* were the most abundant species. The non-native sea urchin *D. setosum* was only recorded in the South Aegean and Levantine Sea.

### 3.3. Fish Biomass

**Figure 5:**
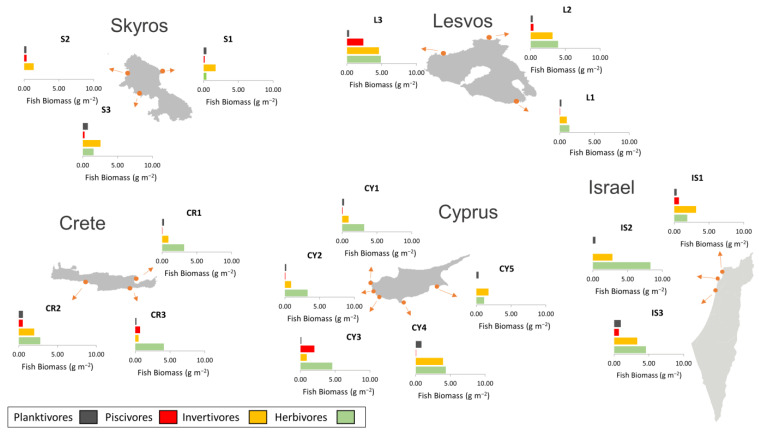
Fish biomass (g m^−2^) estimations for the different fish trophic groups for each study site.

**Figure 6:**
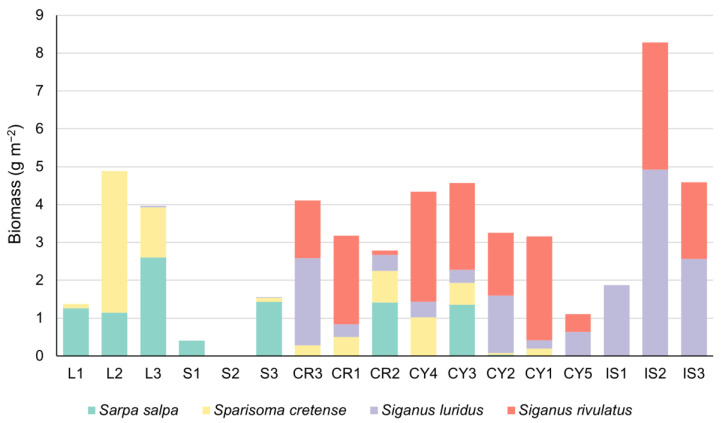
Biomass of herbivorous fish species (g m^−2^) at each study site.

**Figure 7:**
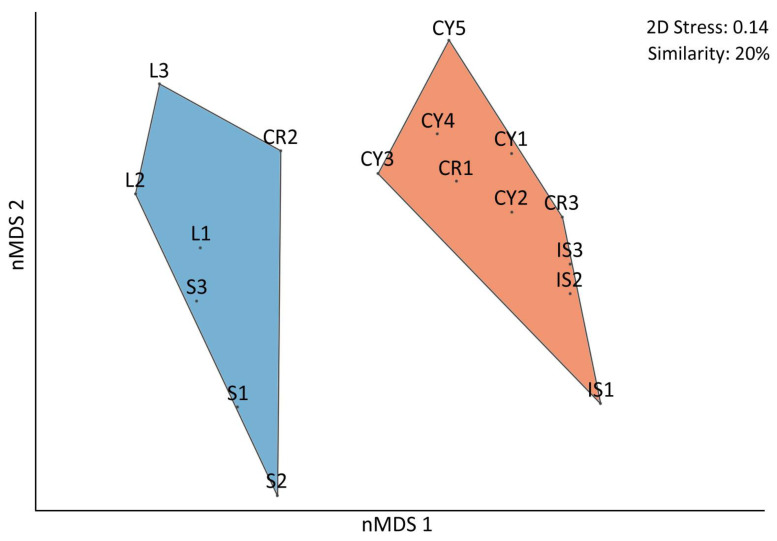
Non-metric multidimensional scaling (nMDS) of fish biomass composition at the surveyed sites, based on the Bray–Curtis dissimilarity (Stress = 0.14). Polygons group sites with 20% similarity based on cluster analysis.

The fish biomass estimations indicated a disparity in herbivore fish biomass between the southern sites of Crete, Cyprus and Israel against the northern sites of Lesvos and Skyros (Figure 5). Specifically, herbivorous fish contributed more significantly to the total fish biomass in the southern sites (ranging from 35 to 75%, with a mean value of 58%) than in the northern sites (ranging from 0 to 52%, with a mean value of 31%). The most abundant herbivorous fishes in the southern sites were *S. rivulatus* and *S. luridus*, followed by *Sparisoma cretense*, whereas in the northern sites, *S. salpa* and *S. cretense* were the dominant recorded fish herbivores (Figure 6). The nMDS analysis also revealed that the fish biomass composition in the southern sites (Crete, Cyprus and Israel) differed from that in the northern Aegean sites (Figure 7), with the exception of site CR2 in Crete, which was clustered with the northern sites.

### 3.4. Modeling Canopy Algae Presence

The most parsimonious GLMM describing canopy algae vegetation included alien herbivores biomass, sea urchin density, and depth. Based on the inspection of scaled residuals analysis, no violations of the model’s assumptions were identified (Figure S2). All independent variables were significantly and negatively correlated with the presence of perennial macroalgae (Table 3).

**Figure 8:**
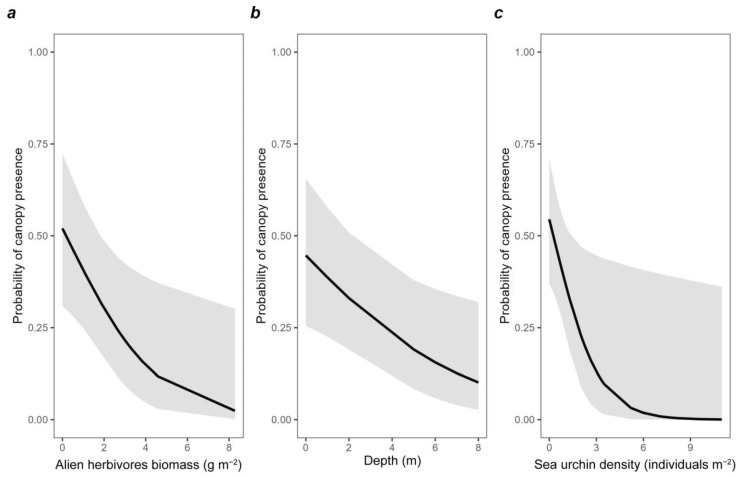
Relation between the probability of the presence of perennial macroalgae and (**a**) alien herbivores fish biomass, (**b**) depth and (**c**) sea urchin density.

The fixed effects accounted for 35.2% (CI = 14.8–80%) of the variance explained, which increased to 36.4% (CI = 17.5–79.9%) when combined with random effects. The sea urchin density contributed the most to the variation in the probability of the presence of macroalgae (semi-partial R^2^ = 0.32 and 95% CI = 0.13–0.79), followed by the depth zone (semi-partial R^2^ = 0.16 and 95% CI = 0.00–0.74) and alien herbivore fish biomass (semi-partial R^2^ = 0.039 and 95% CI = 0.00–0.70) (Figure 8 and Supplementary Table S1; Figure S3).

## 4. Discussion

The present study provides an overview of the environmental status of macroalgal communities in the eastern Mediterranean Sea. Significant differences were found in the bathymetric distribution of macroalgal vegetation between the North Aegean Sea and the South Aegean–Levantine Seas, presumably defined by grazing pressure from sea urchins and invasive fish. A healthier macroalgal community was found in the northern sites, with perennial vegetation coverage observed throughout the depth zones, even peaking at the deeper zones (5 and 8 m). In contrast, an impoverished macroalgae community was recorded in the south, with canopies primarily limited to shallow waters (0–1 m), better protected from grazers, and sparse or absent in deeper waters accessible to grazers.

**Figure 9:**
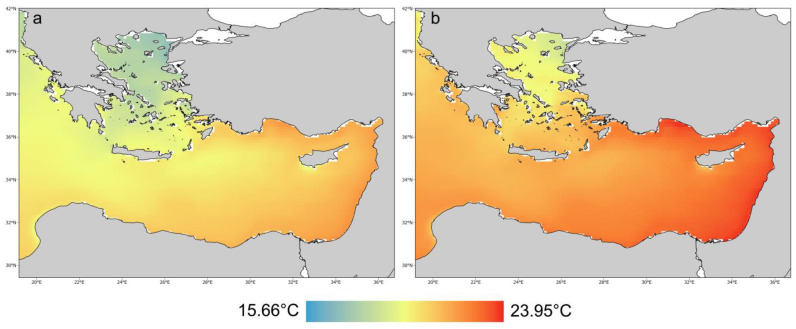
Current (1990–2020) (**a**) and future (2050–2080) (**b**) temperature conditions in the eastern Mediterranean. The values depicted correspond to the mean annual sea surface temperature. Source of simulations: NEMOMED8 climatic model [93] acquired from Med-CORDEX (https://www.medcordex.eu/ accessed on 10 April 2020); future climate data based on projections of IPCC’s Fifth Assessment Report under the “business as usual” climatic scenario RCP8.5.

An important distinction between the northern Aegean and southern sites (Crete, Cyprus and Israel) is the higher contribution of herbivorous fish biomass to the total fish biomass in the south compared to the north. This difference is mainly due to the higher abundance of the two Lessepsian *Siganus* species in the southern sites and their less frequent occurrence in the North Aegean. Other studies have also highlighted the abundant siganid populations in the South Aegean, which decrease in higher latitudes [51,76]. This pattern is consistent with the typical spatial distribution of thermophilic Lessepsian species, with high species richness and abundance in the warmer Levantine region declining towards the colder northern and western Mediterranean regions (Figure 9a) [91,92].

Our results indicate a local collapse of sea urchin populations in the southern sites. This phenomenon has been previously reported along the coast of Israel, where it was found that the continuous sea warming primarily and the intensified competition for food resources from invasive rabbitfish secondarily caused the sea urchin collapse [31,55]. Although the current results only show local sea urchin population collapses in Crete and Cyprus, and a larger-scale study is required to verify the finding regionally, it is a fact that continuous sea warming in the eastern Mediterranean basin and the competitive impact of abundant rabbitfish populations can be detrimental to sea urchins in these islands. The edible *P. lividus* is considered a delicacy in most Mediterranean regions and it is harvested in some areas in the eastern Mediterranean. However, there is no current evidence linking human harvest with the recent local collapse in the Levantine. In contrast, in the North Aegean, where sea temperatures are lower, sea urchins continue to thrive and exert grazing pressure on macroalgae, which is not always sufficient to cause the decline of algal forests. Based on the GLMMs, canopy coverage was negatively correlated with sea urchin density, despite the better status of perennial algal forests in the North Aegean sites. In most North Aegean sites, the highest densities of sea urchins were observed at depths of 1 or 2 m, coinciding with the lowest macroalgal ecological status in this subregion. Similar negative correlations between sea urchins and canopy algae have been previously reported in the Aegean Sea [29,44]. The availability of nutrients is a crucial environmental factor that determines the resilience of macroalgal communities to grazing pressure [94]. The waters of the Aegean Sea show increasing oligotrophy from the north to the south [95], and the more eutrophic and productive waters of the North Aegean could contribute to maintaining a healthier macroalgal ecological status, despite the grazing pressure.

The contrasting vertical distribution of arborescent macroalgae species, specifically *Cystoseira s.l.,* between the northern Aegean and the South Aegean–Levantine sites is presumed to be linked to the abundance of the two main herbivorous groups, sea urchins and fish, as indicated by our modeling results. In the North Aegean, sea urchin populations are most dense at shallow depths and are likely responsible for the low macroalgae cover in some sites. Our analysis did not find a significant relation between native fish biomass and canopy algae presence; native herbivorous fish are likely not abundant enough to reduce arborescent algal cover in the study area significantly. Scientific evidence suggests that *Cystoseira s.l.* forests can flourish even in areas with high *S. salpa* abundance at intermediate depths (~5 m) [46]. In the South Aegean and Levantine sites, sea urchins are rare and thus do not control algal cover at shallow depths. On the other hand, rabbitfish are highly abundant and effective at depths greater than 1 m, removing most erect algae [53]. Some *Cystoseira s.l.* species produce chemical compounds that serve as a defense mechanism against herbivory [96]. However, whether these defense mechanisms work against the invasive *Siganus* species remains unclear. The only constraints that may prevent *Siganus* species from eradicating the remaining arborescent forests of shallow waters in the southeastern sites are the wave action and emergence of shallow parts of the reefs. Recent experiments have shown that sea level rise in the region may expose intertidal macroalgal populations to grazing by rabbitfish, leading to reduced algal cover in very shallow waters in the long term [97]. Further, the species forming the forests may be vulnerable to the effects of ocean warming and ocean acidification [26,27]. For instance, *Gongolaria rayssiae*, a species endemic only to Israel and Lebanon, completely loses its branches in early summer and its optimum temperature matches spring temperatures [98].

The loss of macroalgal forests can have detrimental effects on ecosystems [99,100,101], and this extended loss in the Levantine Sea is a matter of deep concern. Our results suggest that a gradient of change exists from southeast to northwest, primarily due to the combined effects of invasive species and climate change [31]. The changes observed in the southeastern sites are likely a glimpse into the future of the northwestern parts of the Mediterranean (Figure 9b). The alarming rate of change calls for immediate action. Restoration efforts and research must consider region-specific threats, both current and future, to be effective and sustainable. Thorough investigation and support for innovative restoration ideas, coupled with mitigation strategies, such as targeted fishing to control herbivore densities, should be pursued. Some scientists have argued that a large proportion of Mediterranean biota is doomed to local or global extinction. Therefore, conservation planning and management should focus on preserving ecosystem functioning instead of native species, even if functioning is secured by alien species [102,103]. However, current scientific knowledge supports that introduced macroalgae species in the Mediterranean cannot be an equivalent surrogate for *Cystoseira s.l.* forests [104,105]. Mitigation strategies, such as reducing grazing pressure by fishing alien rabbitfish and protecting top predators that control herbivores [106], may effectively restrict the loss of macroalgal forests. However, it is crucial to restore lost habitats through restoration efforts, and continuous ecological monitoring is essential to evaluate the success of these measures.

## 5. Conclusions

Mediterranean ecosystems are undergoing contrasting changes between the colder and warmer parts of the basin. In warmer areas, local biodiversity is collapsing, including the native sea urchin *P. lividus.* Meanwhile, invasive rabbitfish exert strong herbivory pressure on macroalgal communities, limiting *Cystoseira s.l.* forests to the shallowest parts of rocky reefs. In the North Aegean, sea urchins are the primary drivers of the vertical distribution of canopy algae. Given the critical ecological role of *Cystoseira s.l*., the extensive loss of these habitats is alarming. The current state of the Levantine Sea provides valuable insights into the future state of the Mediterranean. We argue that conservation and restoration actions must consider these future threats. The long-term viability of endemic Mediterranean macroalgal forests depends on mitigating the cumulative effects of anthropogenic pressures, including the grazing pressure from invasive rabbitfishes. We also join the calls for the urgent need to reduce global stressors through the mitigation of greenhouse gas emissions and control of bioinvasion vectors to ensure the survival of Mediterranean ecosystems.

## Figures and Tables

**Table 1:** Sea surface temperature (SST) and Secchi transparency depth (Z_SD_) data at each study site for the time period between 2017 and 2022. The spatial resolution for SST data is ~1 × 1 km and 4 × 4 km for the Z_SD_ data. The obtained values serve as an indication of the broader regime in the region; they do not fully describe the study sites as they do not consider the coastal peculiarities and land–sea interactions due to the size of the spatial resolution. Data were obtained from EU Copernicus Marine Service Information (https://marine.copernicus.eu/ accessed on 16 May 2023) [74].

Site	Average SST (°C)	Average Annual Maximum SST (°C)	Average Annual Minimum SST (°C)	Average Z_SD_ (m)
IS1	23.4	30.3	16.8	21.4
IS2	23.4	30.4	16.8	17.8
IS3	23.4	30.4	16.7	20.0
CY1	22.5	29.6	16.5	25.6
CY2	22.4	29.6	16.5	28.6
CY3	22.3	29.2	16.4	27.3
CY4	22.0	28.4	16.6	26.9
CY5	22.5	29.2	16.5	28.2
CR1	20.8	27.2	15.1	25.7
CR2	20.9	26.9	15.3	27.6
CR3	21.2	27.4	15.7	27.3
S1	19.3	27.4	13.9	20.9
S2	19.4	27.3	14.0	22.8
S3	19.3	27.1	13.9	23.2
L1	19.5	25.7	14.9	22.3
L2	18.9	25.6	14.4	20.7
L3	18.9	25.4	14.5	22.4

**Table 2:** Status scores of the macroalgal communities, adapted from Thibaut et al. [75]. Scores were determined based on the coverage of arborescent perennial species (*Cystoseira s.l.* and *Sargassum* spp.), shrubby and turf/encrusting alga.

Status	4 (Very Good)	3 (Good)	2 (Moderate)	1 (Low)	0 (Very Low)
Cover Type	Arborescent perennial ≥50%	Arborescent perennial 5 to 50%	Shrubby ≥50%	Shrubby 5 to 50%	Turf Encrusting

**Table 3:** Results of the most parsimonious GLMM describing the presence of perennial macroalgae.

Predictors	β Estimates	Odds Ratios	Z-Value	*p*-Value
(Intercept)	1.46	4.30	2.15	0.031
Alien Herbivores	−0.46	0.63	−2.25	0.024
Depth	−0.25	0.78	−2.33	0.020
Sea urchins	−0.69	0.50	−2.06	0.039
N site	17			
Observations	76			
Marginal R^2^/Conditional R^2^	0.352/0.364			
Log-likelihood	−40.291			

## Data Availability

The data presented in this study are available on request from the corresponding author.

## Supplementary Materials

The following supporting information can be downloaded at: https://www.mdpi.com/article/10.3390/biology12060763/s1, Figure S1: Photo-quadrat samples taken at stations CY2 and S2 and the quadrat scores based on the reef-EBQI index; (a) CY2 at the 0-0.5 m depth zone quadrat scored as 4, (b) CY2 at the 2 m depth zone quadrat scored as 1, (c) CY2 at the 8 m depth zone quadrat scored as 0, (d) S2 at the 0-0.5 m depth zone scored as 2, (e) S2 at the 2 m depth zone scored as 3 and (f) S2 at the 8 m depth zones scored as 4. Note that quadrats in pictures (a) and (d) are 25 × 25 cm and all others are 50 × 50 cm; Figure S2: DHARMa diagnostics on the left Q-Q plot with added tests for corrected distribution (Kolmogorov-Smirnov test), dispersion and outliers. On the right residuals are plotted against the predicted value with quantile regression lines; Figure S3: Forest plot for the best model displaying the estimated semi-partial R2 and related 95% confidence intervals for different independent variables and combinations; Table S1: Generalized mixed models built with a binomial distribution and logit link function. Candidate models are ranked by the Akaike Information Criterion corrected for small samples (AICc).
