# Supplementary material for: Invasive Fish and Sea Urchins Drive the Status of Canopy Forming Macroalgae in the Eastern Mediterranean
Source: Biology (Basel). 2023 May 24;12(6):763. doi: 10.3390/biology12060763 (PMC10295672; doi:10.3390/biology12060763)
Supplement: Supplementary file 1 [file biology-12-00763-s001.zip › Supplementary material.pdf]

Table S1. Generalized mixed models built with a binomial distribution and logit link function. Candidate models are ranked by the Akaike Information Criterion corrected for small samples (AICc).

Model ID	Intercept	Alien H	Depth	Native H	Sea urchins	df	Log-Likelihood	AICc	ΔAICc	Weight
mod4	1.459	-0.4567	-0.246		-0.6893	5	-40.291	91.4	0	0.527
mod1	1.494	-0.4647	-0.2457	-0.01883	-0.6893	6	-40.287	93.8	2.35	0.163
mod13	0.5418		-0.2537		-0.6204	4	-42.868	94.3	2.86	0.126
mod7	0.4362	-0.3826			-0.5117	4	-43.558	95.7	4.24	0.063
mod9	0.3097		-0.2546	0.2222	-0.6328	5	-42.482	95.8	4.38	0.059
mod3	0.4827	-0.3928		-0.02406	-0.5128	5	-43.551	98	6.52	0.02
mod14	-0.3739				-0.3949	3	-46.003	98.3	6.9	0.017
mod11	-0.5602			0.1774	-0.4105	4	-45.652	99.9	8.43	0.008
mod15	-0.2131		-0.1546			3	-47.274	100.9	9.44	0.005
mod0	-0.6822					2	-48.749	101.7	10.22	0.003
mod5	0.03222	-0.152	-0.1473			4	-46.762	102.1	10.65	0.003
mod8	-0.3935	-0.1636				3	-48.106	102.5	11.11	0.002
mod10	-0.3045		-0.1537	0.07316		4	-47.197	103	11.52	0.002
mod12	-0.7776			0.07875		3	-48.654	103.6	12.2	0.001
mod2	0.06353	-0.1584	-0.1471	-0.01667		5	-46.758	104.4	12.94	0.001
mod6	-0.3566	-0.1711		-0.01934		4	-48.101	104.8	13.33	0.001

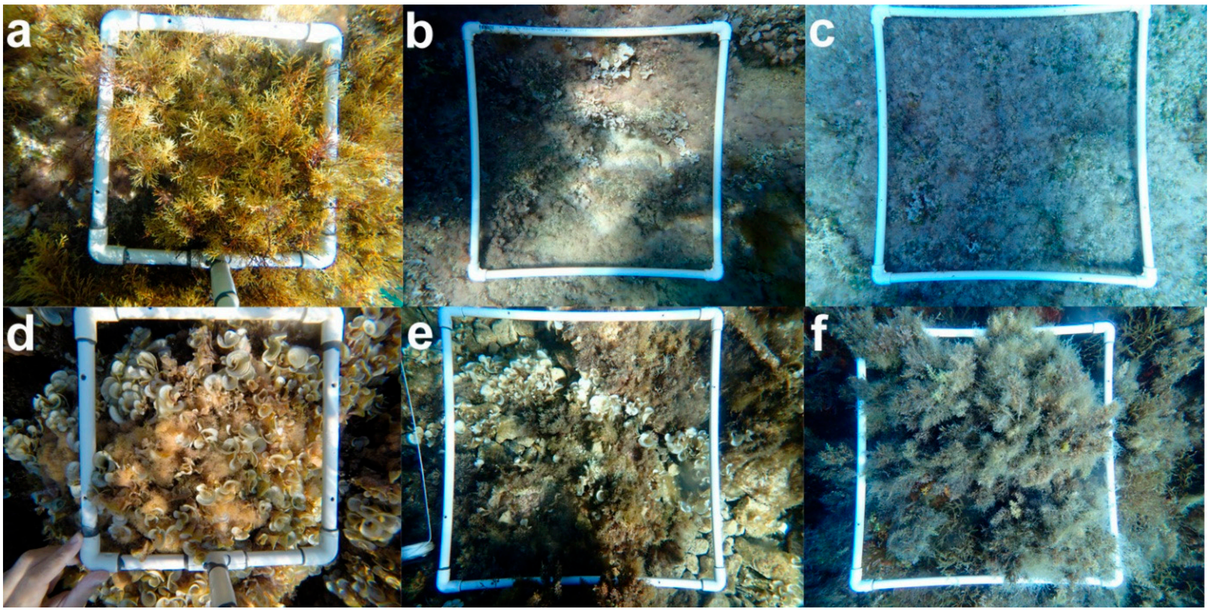

Figure S1. Photo-quadrat samples taken at stations CY2 and S2 and the quadrat scores based on the reef-EBQI index; (a) CY2 at the 0-0.5 m depth zone quadrat scored as 4, (b) CY2 at the 2 m depth zone quadrat scored as 1, (c) CY2 at the 8 m depth zone quadrat scored as 0, (d) S2 at the 0-0.5 m depth zone scored as 2, (e) S2 at the 2 m depth zone scored as 3 and (f) S2 at the 8 m depth zones scored as 4. Note that quadrats in pictures (a) and (d) are 25 x 25 cm and all others are 50 x 50 cm.

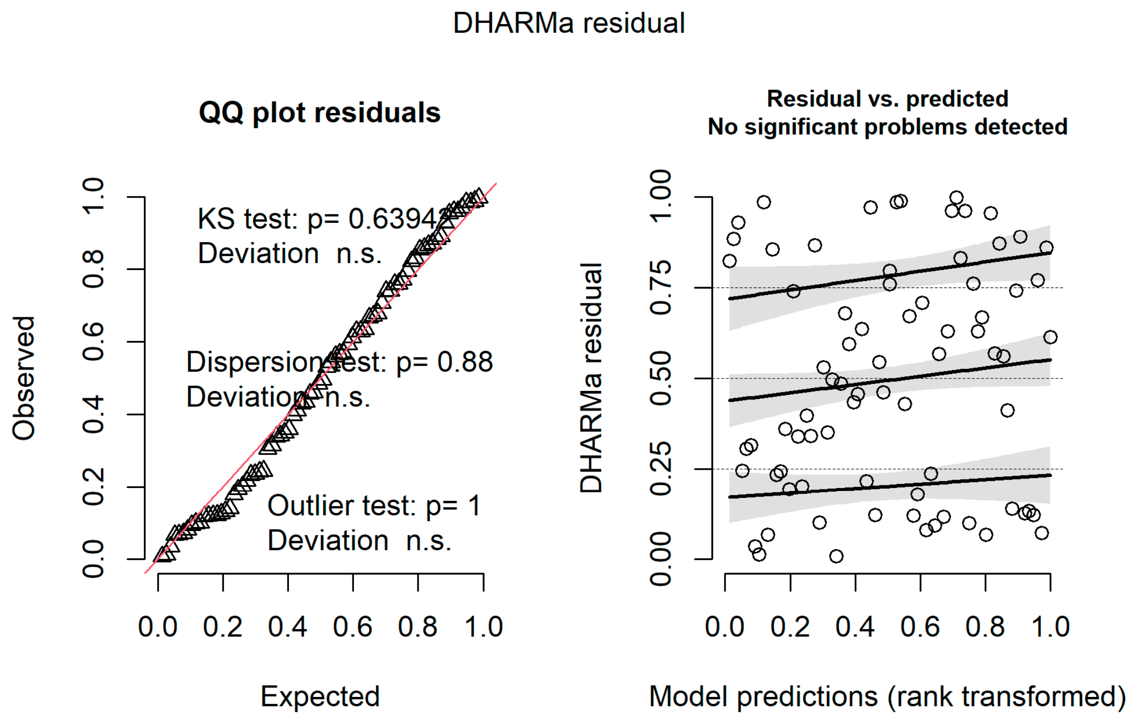

Figure S2. DHARMa diagnostics on the left Q-Q plot with added tests for corrected distribution (Kolmogorov-Smirnov test), dispersion and outliers. On the right residuals are plotted against the predicted value with quantile regression lines.

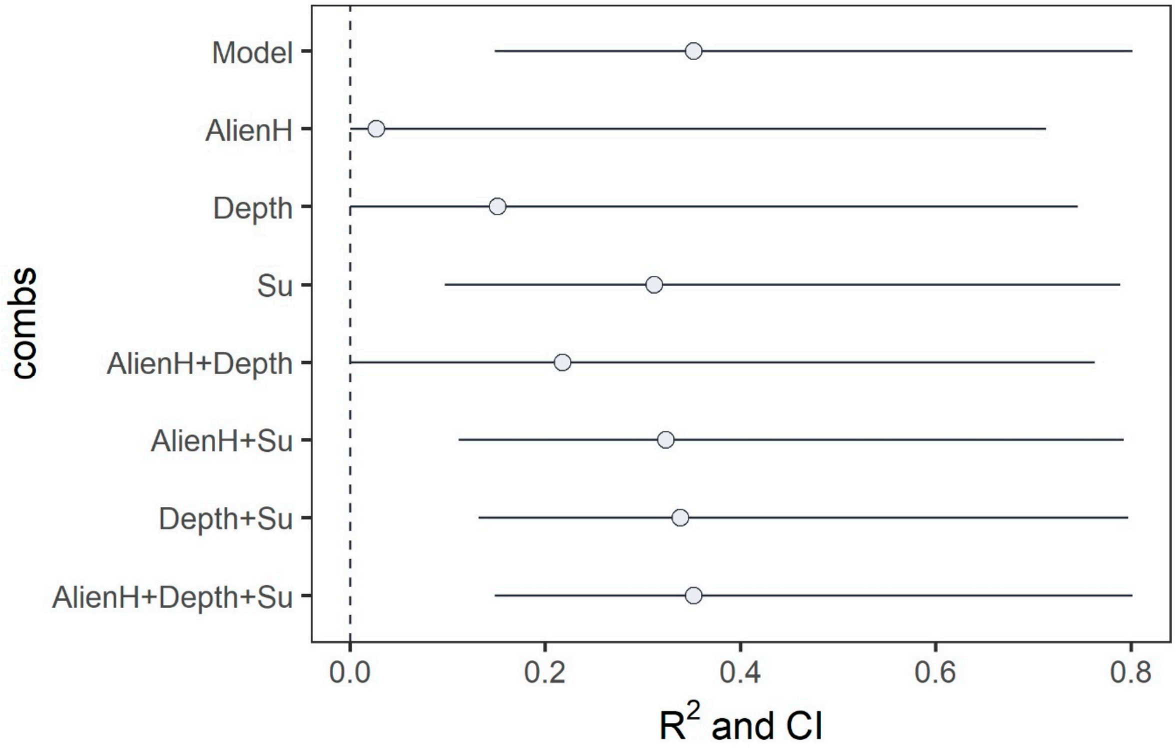

Figure S3. Forest plot for the best model displaying the estimated semi-partial R^2 and related 95% confidence intervals for different independent variables and combinations.
